# Hepatitis B and human immunodeficiency virus infections within correctional facilities in Ghana

**DOI:** 10.1371/journal.pone.0293009

**Published:** 2023-11-03

**Authors:** Kwamena W. C. Sagoe, Kyeremeh Atuahene, Angela N. A. Ayiku, Prince J. Pappoe-Ashong, Isaac Boamah, Holger Till, Francis Selorm Hagbe, Isaac Kofi Egyire, Matilda Nyampong, Stephen Ayisi Addo, Adom Manu, Charles L. Noora, Millicent Tetteh, Augustine Ankomah, Richard Adanu

**Affiliations:** 1 Department of Medical Microbiology, University of Ghana Medical School, College of Health Sciences, University of Ghana, Accra, Ghana; 2 Ghana AIDS Commission, Accra, Ghana; 3 Deutsche Gesellschaft für Internationale Zusammenarbeit GmbH, Accra, Ghana; 4 Ghana Prisons Service, Accra, Ghana; 5 National AIDS/STI Control Programme, Accra, Ghana; 6 Department of Population, Family and Reproductive Health, School of Public Health, College of Health Sciences, University of Ghana, Accra, Ghana; 7 Department of Epidemiology and Disease Control, School of Public Health, College of Health Sciences, University of Ghana, Accra, Ghana; University of Cincinnati College of Medicine, UNITED STATES

## Abstract

Previous studies have suggested high Immunodeficiency Virus (HIV) and hepatitis B virus (HBV) prevalence in prisons in Ghana. However, this study was part of a nationally representative bio-behavioural survey and determined the prevalence of HIV and HBV among prison inmates and identified factors associated with these infections. Both biomedical and behavioural data were collected from a total of 2,443 prison inmates from 19 prison stations during 2013 in Ghana; 12 male prisons and 7 female prisons selected across the country. The national HIV screening algorithm was used for HIV testing while two rapid detection tests were used to confirm HBV infections. HIV and HBV prevalence among prisoners in Ghana were approximately 2.34% and 12.38% respectively. Only 5 inmates, had co-infection with both viruses. The prevalence of HIV was significantly lower among male inmates (1.5%) compared to the female inmates (11.8%). Age, sex, and marital status, were significantly associated with both HIV and HBV infections. However, BMI category, IDU, and time spent in prison were associated with HIV infections. The educational level was significantly associated with HBV infections. After binary logistic regression, being female (AOR: 0.18, 95% CI: 0.07–0.45, p<0.001) and having a stay of 5 years or more (AOR: 0.07, 95% CI: 0.01–0.60, p = 0.016), increased the risk of having HIV infection. While, those with no formal education (AOR: 0.65, 95% CI: 0.45–0.95, p = 0.024) and are underweight (AOR: 0.51, 95% CI: 0.27–0.99, p = 0.046), were more likely to have HBV infection. Forced penetrative sex may be a problem in the prisons. The need to have and strengthen an integrated screening, treatment and vaccination plan for the prison is emphasized. The prison does not serve as an exceptionally high risk to the general population. The findings support a critical look at the issue of forced penetrative sex in the prisons.

## Introduction

Correctional facilities worldwide are considered environments for fast and uncontrolled spread of blood borne viruses (BBV) such as Human immunodeficiency virus (HIV) and Hepatitis B virus (HBV) and many other infectious diseases due to specific risk practices, poor health care and living conditions [1–3]. Globally, there is a gross disproportion in rates of infection among isolated populations such as seen in prisons and the general population [4–7]. Many studies in sub-Saharan Africa and in Ghana have revealed that the prevalence of BBVs in the general population is lower than that among high-risk groups [3,8]. Those at high-risk include blood or tissue donors, haemodialysis patients, HIV-positive patients, household members or sexual contact of infected individuals, individuals with conditions that may require immunosuppressive or immune-modifying therapy, infants born to HBV-infected mothers, injection drug users, men who have sex with men and pregnant women [9–15]. Among these high-risk groups injecting drug users (IDU) and Men who have sex with Men (MSM) are said to be more common among incarcerated populations [2,16–19]. It is well known that behaviours such as sex, tattooing and injection drug use are illegal at the prison sites but these are the most prevalent intra-prison high-risk behaviours that put inmates at risk of blood-borne infections including HIV and HBV which may account for the higher prevalence in the prisons [19].

International data suggests that the prevalence of HIV among prisoners may be three to fifty times higher than in the general adult population [8,20–23]. Relatedly, HBV prevalence within prisons in Ghana are higher than the national prevalence [3]. According to statistics for the year 2013 from the Ghana Prisons Service, there are forty-two (42) prison establishments nationwide who at the time had a population of approximately 13,908 inmates. This consists of approximately 10,886 convicted and 3,023 prisoners who were yet to be convicted (on remand), the majority of which were men (10,725 convicted and 2,966 on remand) [24]. There were only about 217 women and 117 juveniles [24].

Antiviral therapy for both HIV and HBV, as well as vaccines to prevent HBV disease, are currently available in Ghana. It is therefore important to identify risk behaviours associated with infections within correction facilities in order to develop protocols and also to link infected prisoners to therapy.

This study was part of data collected from a behavioral and health survey conducted in Ghana from the month of February to March in 2013. The prevalence and risk factors associated with HIV and HBV infections were determined, and implications for the treatment and eradication of both infections discussed.

## Methods and materials

### Study design

A cross-sectional design employing quantitative methods were used to assess the situation of HIV and HBV infections as well as other key noncommunicable illnesses among prison inmates. Interviewer-administered standardised behavioural questionnaires for inmates were used to assess the situation in the prison and determine potential risk factors, at one point in time, relying on participant’s recall of their behaviour before and during imprisonment. The study integrated results from the biological tests with the behavioural questionnaires covering inmates’ knowledge, attitudes, behaviours and environmental factors.

### Sampling and administration of questionnaire

The study consisted of male and female prison inmates on remand, awaiting trial, convicted and sentenced. Sampling was done at all locations and for all categories of male prisons (one Maximum security prison, one Medium security prison, eight Central prisons, fifteen Local prisons and ten Camp prisons with lower levels of security). For the females, only one Medium security prison, and six local prisons were included. A census was conducted of all 260 female inmates scattered across seven prison locations who consented to be part of the study. A stratified random sample was taken of the male prison inmates. All inmates were 18 years (age of consent) and above.

Nationally, the sample size required for a simple random sample of 13,500 prisoners is 1,842 using 1% standard error. Adjusting for the provision of an estimated 80% response rate (20% refusal rate by participants), the sample size was increased to a total of 2,238 male inmates. A total of 2,443 prisoners were successfully enrolled and interviewed, comprising 205 female and 2,238 male inmates. Proportionate sampling relative to the population of each category was conducted and respective sampling intervals were computed and applied appropriately to the various categories (Table 1).

**Table 1:** Selected prison sites by categories with their respective sample sizes.

Prison Name/type		Total population by station	Proposed Sample		Actual Sample
**Maximum Security Prison**					
1		264	45		49
**Medium Security Prison**					
1		3,570	611		652
**Central Prisons**					
-1		1,940	456		437
2		852	200		199
3		274	65		67
4		200	47		65
5		370	87		126
**Local Prisons**					
1		185	172		117
2		161	150		80
3		264	264		208
**Camps Prisons**					
1		200	85		104
2		330	140		141
**TOTAL MALES**		**8,610**	**2303**		**2238**
**Females**					
1	133			98	
2	15			11	
3	26			23	
4	38			26	
5	20			17	
6	18			16	
7	9			7	
**TOTAL FEMALES**	**259**			**205**	

At each prison station, the purpose and the various procedures involved in the study was explained to all selected participants. Thereafter, interviews were conducted between 20–25 minutes in the open but in isolation from other participants and interviewers to ensure privacy and confidentiality. The questionnaire consisted of seven sections as follows: Socio-demographic characteristics.; prison environment; health in prison; HIV risk—blood contact; HIV risk—injecting drug use; HIV risk—sexual contact; and HIV/AIDS knowledge and attitudes.

The interview was administered in either English or appropriate local language. At the end of each interview, participants were issued a “Counselling and Testing Card” to enable tracking of participants through all testing procedures.

The questionnaire was pre-tested involving 20 inmates at Winneba Prison, which was not part of the sample. The pilot study was to assess whether the questions were clear and easy to understand and also examine whether the listed categories of answers catered for all possible responses. Amendments were made to the questionnaire subsequently.

At each prison station, the purpose and the various procedures involved in the study was explained to all selected participants. Thereafter, interviews were conducted between 20–25 minutes in the open but in isolation from other participants and interviewers to ensure privacy and confidentiality. The questionnaire consisted of seven sections as follows: Socio-demographic characteristics, prison environment, health in prison, HIV risk—blood contact, HIV risk—injecting drug use, HIV risk—sexual contact and HIV/AIDS knowledge and attitudes.

The interview was administered in either English or appropriate local language. At the end of each interview, participants were issued a “Counselling and Testing Card” to enable tracking of participants through all testing procedures. The questionnaire was pre-tested with 20 inmates at Winneba Prison, which was not part of the sample. The pilot study was to assess whether the questions were clear and easy to understand and also examine whether the listed categories of answers catered for all possible responses. Amendments were made to the questionnaire subsequently.

### Diagnostic procedures and counselling

Well trained biomedical personnel and a professional HIV/AIDS counsellor for testing and counselling respectively carried out this component. Pre- and post-counselling was done on one-on-one basis.

The weight and height were measured using standard anthropometric procedures for measuring adults and BMI was calculated. Thereafter, 3–5 ml sample of blood was taken using a 5ml EDTA Vacutainer tube. The blood was thoroughly mixed by gently swirling and turning tubes upside down 15 times and placed in a rack. Following manufactures instructions as described in the test protocol, HIV was confirmed using the First-Response HIV1/2 (Transnational Technologies Inc., Norwich, UK) as an initial test, and OraQuick Rapid HIV1/2 antibody test (OraSure Technologies Inc., Bethlehem, PA) as a supplemental test. Plasma from the EDTA tubes was used to test for the Hepatitis B surface antigen (HBsAg) was detected concurrently with Wondfo One Step HBsAg test (Guangzhou, Wondfo Biotech. Co. Ltd.) and Core ^TM^ HBsAg (Core Diagnostics, Birmingham, UK). In both algorithms, the two screening tests had to be reactive to confirm HIV and HBV infections.

After rapid testing in the prisons, blood samples in their vacutainer tubes were transported on ice packs to the nearest Regional/District–Ghana Health Service Laboratory (GHS lab). Aliquots of plasma were stored at 2–8°C for 3–5 days after which they were transported on ice packs to the Clinical Virology Laboratory, Department of Medical Microbiology, University of Ghana, for permanent storage at –20°C.

### Data management and statistical analysis

Completed questionnaires were reviewed twice a week by field supervisors to ensure consistency and check for any reoccurring errors. The results from the diagnostic tests were recorded directly onto the anonymous, coded questionnaires to ensure that there was no misallocation between the behavioural and biological data. Data from the questionnaires were entered and analysed by the Statistical Package for Social Sciences (SPSS) version 18.0 software (IBM Corp., Armonk, NY). The data entry was independently verified and cleaned by a second investigator.

Bi-variate analysis was conducted to determine whether there were significant associations between inmates’ HIV, HBV, BMI, and their gender, age, education, length of imprisonment, type of prison, risk behaviours, and knowledge of HIV/AIDS.

Given the risk that bivariate associations can be confused by confounding factors, a multivariate analysis using logistic regression was also carried out to establish ultimately, which factors were significantly associated with inmates’ HIV and HBV prevalence. The Pearson’s chi-square test was used to assess the association between prisoners’ characteristics and their HIV and HBV status. The simple binary logistics regression model was used to estimate the crude odds ratios of both HIV and HBV positivity across the various characteristics of the prisoners. The multiple binary logistic regression model was then used to estimate the adjusted odds ratios of HIV and HBV positivity across the various characteristics of the prisoners.

The Fischer’s exact chi-square test was used to assess the association between inmate characteristics and prevalence of both HIV and HBV positivity among the prisoners. The frequency and percentage distribution of both HIV and HBV positivity were described across the various categories of the characteristics of the inmates.

### Ethics

Approval for the study was obtained from the Noguchi Memorial Institute for Medical Research Institutional Review Board (NMIMR- 007/11-12) and the Ghana Health Service Ethical Review Committee (GHS-ERC 05/9/11). Prior to the study, two levels of written signed (or thumbprint) consent for the survey and blood specimen banking were obtained. During the period of obtaining informed written consent from the inmates, they were made to understand that their participation was voluntary and that they were free to opt out of the study.

## Results

### Characteristics of study participants

A total of 2443 prisoners were interviewed in the study with a median age of 31 years (Interquartile Range (IQR): 26 to 40 years). Most of the prisoners were between the age range of 20–29 years (n = 991, 40.6%), with 124 (5.1%) above 59 years. Two thousand two hundred and thirty-four (91.6%) of the prisoners were males, 428 (17.5%) had no formal education whilst 94 (3.9%) had college or university level education. Three-quarter (n = 1867, 76.4%) were Christians, 1035 (42.4%) had never married whilst 1129 (46.2%) were currently married. Majority (n = 2063, 84.5%) were convicted prisoners whilst 380 (15.6%) were on remand.

Majority of the prisoners had normal weight (72.5%, n = 1772), 643 (26.3%) were hypertensive, 1694 (69.3%) had ever use drugs, and about a third (32.0%) had been in prison for the current sentence or remand for less than a year. Detailed characteristics of the prisoners are shown in Table 2.

**Table 2:** Characteristics of study participants.

Variables	Frequency (N = 2443)	Percentage
**Age, median (IQR)**	31 (26, 40)	
**Age group**		
18–19	69	2.82
20–29	991	40.56
30–39	732	29.96
40–49	365	14.94
50–59	161	6.59
>59	124	5.08
Non response	1	0.04
**Sex**		
Female	205	8.39
Male	2238	91.61
**Educational level**		
No education	428	17.52
Primary	781	31.97
Junior high	767	31.4
Senior high	373	15.27
College/university	94	3.85
**Religion**		
Christian	1867	76.42
Muslim	516	21.12
Traditionalist	24	0.98
No religion	35	1.43
No response	1	0.04
**Marital status**		
Single (never married)	1035	42.37
Currently married	1129	46.21
Divorced/separated/widowed	268	10.97
No response	11	0.45
**Type of prisoner**		
Convicted	2063	84.45
On Remand /awaiting trial	380	15.55
**BMI, median (IQR)**	22.91 (21.30, 24.86)	
**BMI category**		
Underweight	86	3.52
Normal	1772	72.53
Overweight	481	19.69
Obese	104	4.26
**Prison type**		
Agricultural Camp	325	13.3
Central Prison	981	40.16
Local Prison	337	13.79
Maximum security	49	2.01
Medium security	751	30.74
**Injecting Drug Use (IDU)**		
No	743	30.41
Yes	1694	69.34
Non-response	6	0.25
**Time in prison for current sentence/remand**		
<1 year	782	32.01
1–5 years	1311	53.66
>5 years	350	14.33
**Region**		
Ashanti	567	23.21
B. Ahafo	215	8.8
Central	177	7.25
Eastern	879	35.98
Gt Accra	140	5.73
Northern	74	3.03
Upper west	65	2.66
Volta	225	9.21
Western	101	4.13

### Prevalence of HIV and HBV among inmates

Among the 2,436 prisoners who had HIV test results, 58 (2.38%; CI 95%, 1.81% to 3.07%). Furthermore, 313 (12.82%; CI 95%, 11.52% to 14.21%) of the 2,442 prisoners that had HBV test results were positive. Only 5 (0.21%; CI 95%, 0.07% to 0.48%) of the 2,435 who screened for both HIV and HBV had coinfection.

### HIV status and associated characteristics

The frequency and percentage positivity and negativity were also described across the prisoners’ characteristics (Table 3).

**Table 3:** Association between characteristics and HIV status.

	HIV status				
Variables	Total	Negative (%)	Positive (%)	χ^2^-value	P-value
Overall	2435	2377 (97.62)	58 (2.38)		
**Age group in years**				12.24	0.032^+^
18–19	69	68 (98.55)	1 (1.45)		
20–29	989	976 (98.69)	13 (1.31)		
30–39	729	709 (97.26)	20 (2.74)		
40–49	364	348 (95.60)	16 (4.40)		
50–59	160	155 (96.88)	5 (3.13)		
>59	124	121 (97.58)	3 (2.42)		
**Sex**				84.94	<0.001*
Female	203	179 (88.18)	24 (11.82)		
Male	2233	2199 (98.48)	34 (1.52)		
**Educational level**				2.58	0.631^+^
No education	428	417 (97.43)	11 (2.57)		
Primary	776	754 (97.16)	22 (2.84)		
Junior High	767	754 (98.31)	13 (1.69)		
Senior High	371	362 (97.57)	9 (2.43)		
College/University	94	91 (96.81)	3 (3.19)		
**Religion**				1.11	0.774^+^
Christian	1861	1815 (97.53)	46 (2.47)		
Muslim	515	505 (98.06)	10 (1.94)		
Traditionalist	24	24 (100.00)	0 (0.00)		
No religion	35	34 (97.14)	1 (2.86)		
**Marital status**				15.73	<0.001*
Single (never married)	1034	1021 (98.74)	13 (1.26)		
Currently married	1125	1094 (97.24)	31 (2.76)		
Divorced/separated/widowed	266	252 (94.74)	14 (5.26)		
**Type of prisoner**				1.28	0.258*
Convicted	2061	2015 (97.77)	46 (2.23)		
On Remand /awaiting trial	375	363 (96.80)	12 (3.20)		
**BMI category**				9.26	0.026^+^
Underweight	86	85 (98.84)	1 (1.16)		
Normal	1766	1727 (97.79)	39 (2.21)		
Overweight	480	469 (97.71)	11 (2.29)		
Obese	104	97 (93.27)	7 (6.73)		
**Prison type**				7.57	0.109^+^
Agricultural Camp	325	321 (98.77)	4 (1.23)		
Central Prison	978	952 (97.34)	26 (2.66)		
Local Prison	337	334 (99.11)	3 (0.89)		
Maximum security	49	47 (95.92)	2 (4.08)		
Medium security	747	724 (96.92)	23 (3.08)		
**Injecting Drug Use (IDU)**				25.07	<0.001*
No	740	705 (95.27)	35 (4.73)		
Yes	1690	1667 (98.64)	23 (1.36)		
**Time in prison for current sentence/remand**				9.12	0.010^+^
<1 year	776	749 (96.52)	27 (3.48)		
1–5 years	1310	1281 (97.79)	29 (2.21)		
>5 years	350	348 (99.43)	2 (0.57)		
**Region**				11.38	0.181^+^
Ashanti	565	556 (98.41)	9 (1.59)		
B. Ahafo	214	204 (95.33)	10 (4.67)		
Central	177	171 (96.61)	6 (3.39)		
Eastern	875	851 (97.26)	24 (2.74)		
Gt Accra	140	138 (98.57)	2 (1.43)		
Northern	74	74 (100.00)	0 (0.00)		
Upper west	65	64 (98.46)	1 (1.54)		
Volta	225	222 (98.67)	3 (1.33)		
Western	101	98 (97.03)	3 (2.97)		

*p-values obtained with Pearson’s Chi-squared test.

+p-values obtained with Fisher’s exact test.

From the Pearson’s chi-square test, age was found to be significantly associated with the HIV status of the prisoners (χ^2^ = 12.24, p = 0.032) with HIV positivity of 1.5% (n = 1/69) among those age 18–19 years, 1.3% (n = 13/989) among those aged 20–29 years, 2.7% (n = 20/729) among 30–39 years old, 4.4% (n = 16/364) among 40–49 years old, 3.1% (n = 5/160) among 50–59 years old and 2.4% (n = 3/124) among those 60 years and above.

Prevalence of HIV was significantly higher among female prisoners (11.8%, n = 24/203) than among male prisoners (1.52%, n = 34/2233) from Pearson’s chi-square (χ^2^ = 84.94, p<0.001). HIV positivity was significantly higher among those who were divorced, separated or widowed (5.3%, n = 14/266) compared to those who had never married (1.3%, n = 13/1034) or those who were currently married (2.8%, n = 31/1125) from the Pearson’s chi-square test (χ^2^ = 15.73, p<0.001).

Prevalence of HIV was also significantly higher among obese prisoners (6.7%, n = 7/104) compared to those underweight (1.2%, n = 1/86), those with normal weight (2.2%, n = 39/1766) and those who were overweight (2.3%, n = 11/480) from the Pearson’s chi-square test (χ^2^ = 9.26, p = 0.026). Prevalence of HIV was significantly higher among prisoners who have never used drugs (4.7%, n = 35/740) compared to those who had ever used drugs (1.4%, n = 23/1690) from the Pearson’s chi-square test (χ^2^ = 25.07, p<0.001).

Prevalence of HIV was significantly higher among prisoners who have been in prisons for less than a year for their current conviction or remind (3.5%, n = 27/776) compared to those with 1–5-year stay (2.2%, n = 29/1310) and those with more than 5 years stay (0.6%, n = 2/350) from the Pearson’s chi-square test (χ^2^ = 9.12, p = 0.010) (Table 3).

### Binary logistic regression model of factors associated with HIV status among inmates

From the multiple binary logistic regression model, the adjusted odds of HIV positivity was 82% (AOR: 0.18, 95% CI: 0.07–0.45, p<0.001) less among male inmates than females. Also, for prisoners who have currently been in prison for less than a year for their current sentence or remand, the adjusted odds of HIV positivity was 93% (AOR: 0.07, 95% CI: 0.01–0.60, p = 0.016) less among those who have been in prison for more than 5 years. Overall, the time served for current sentence or remand was found to be a significant factor associated with HIV status of prisoners (p = 0.037).

### HIV status and sexual behavioural characteristics

Sexual risk behavioural experiences of inmates before imprisonment such as casual sex partnerships and sex with commercial sex workers (CSW) and forced penetrative sex within prison with other inmates were examined with HIV prevalence among prisoner (Table 4).

**Table 4:** Sexual behavioural variables by HIV status.

Characteristic	Total	% HIV Positive	*p*-Value
**Casual sex partners before prison**			*p* = 0.210
Yes	1351	2.0	
No	1082	2.8	
**Number of casual sex partners in the past 12 months**			*p* = 0.197
Single partner	675	2.5	
2 or more partners	657	1.5	
**Condom use during casual sex**			*p* = 0.228
Never use condom	790	1.8	
Inconsistence use	372	3.0	
Consistence use	131	0.8	
**Sex with CSW before prison**			*p* = 0.095
Yes	516	1.4	
No	1916	2.6	
**Frequency of sex with CSW**			*p =* 0.633
At least once a week	121	0.8	
Few times a month	200	2.0	
Few times a year	179	1.1	
**Frequency of condom use with CSW**			*p* = 0.810
Never used condom	128	0.8	
Inconsistence use	97	1.0	
Consistence use	264	1.5	
**Heard of inmates being forced to have penetrative sex**			*p* = 0.043
Yes	1281	1.6	
No	1128	3.1	
Don’t know	19	5.3	
**Frequency of forced penetrative sex**			*p* = 0.071
Every few days	328	0.3	
Every few weeks	344	2.0	
Every few months	564	2.3	

HIV prevalence was 2.8% among those who had never engaged in casual sex partners compared with those who reported casual sex (2%). Among those who reported casual sex, prevalence was highest (3%) amongst those who reported inconsistent condom use.

Similarly, HIV prevalence is higher (2.6%) among inmates who reported they had never engaged in sex with commercial workers compared to 1.4% among those who had ever done so. There was no statistically significant difference in condom use among inmates who had ever had sex with CSWs.

### HBV status and associated characteristics

The prevalence of HBV was highest in inmates between the ages of 20–29 years (14.8%, n = 147/991) and lowest among those above 59 years (7.3%, n = 9/124). There were significant differences in prevalence between the various age groups (χ^2^ = 11.39, p = 0.044). The prevalence of HBV was significantly higher among male inmates (13.3%, n = 297/2237) compared to females (7.8%, n = 16/205; χ^2^ = 5.03, p = 0.025). There were varying degrees of prevalence among the various levels of education (χ^2^ = 11.95, p = 0.018). HBV positivity was significantly higher among inmates who had never married (14.0%, n = 145/1035) compared to those who were currently married (12.9%, n = 146/1129) and those who were divorced, separated or widowed (7.5%, n = 20/267; χ^2^ = 8.12, p = 0.017). Details of the association between characteristics and HBV status are shown in Table 5.

**Table 5:** Association between characteristics and HBV status.

	HBV status				
Variables	Total	Negative (%)	Positive (%)	χ^2^-value	P-value
Overall	2441	2128 (87.18)	313 (12.82)		
**Age group**				11.39	0.044*
18–19	69	60 (86.96)	9 (13.04)		
20–29	991	844 (85.17)	147 (14.83)		
30–39	732	640 (87.43)	92 (12.57)		
40–49	364	320 (87.91)	44 (12.09)		
50–59	161	149 (92.55)	12 (7.45)		
>59	124	115 (92.74)	9 (7.26)		
**Sex**				5.03	0.025*
Female	205	189 (92.20)	16 (7.80)		
Male	2237	1940 (86.72)	297 (13.28)		
**Educational level**				11.95	0.018*
No education	428	363 (84.81)	65 (15.19)		
Primary	781	702 (89.88)	79 (10.12)		
Junior High	766	672 (87.73)	94 (12.27)		
Senior High	373	314 (84.18)	59 (15.82)		
College/University	94	78 (82.98)	16 (17.02)		
**Religion**				6.60	0.086^+^
Christian	1866	1642 (88.00)	224 (12.00)		
Muslim	516	433 (83.91)	83 (16.09)		
Traditionalist	24	21 (87.50)	3 (12.50)		
No religion	35	32 (91.43)	3 (8.57)		
**Marital status**				8.12	0.017*
Single (never married)	1035	890 (85.99)	145 (14.01)		
Currently married	1129	983 (87.07)	146 (12.93)		
Divorced/separated/widowed	267	247 (92.51)	20 (7.49)		
**Type of prisoner**				0.20	0.651*
Convicted	2062	1795 (87.05)	267 (12.95)		
On Remand /awaiting trial	380	334 (87.89)	46 (12.11)		
**BMI category**				3.41	0.332*
Underweight	86	70 (81.40)	16 (18.60)		
Normal	1772	1544 (87.13)	228 (12.87)		
Overweight	480	425 (88.54)	55 (11.46)		
Obese	104	90 (86.54)	14 (13.46)		
**Prison type**				4.22	0.377*
Agricultural Camp	324	278 (85.80)	46 (14.20)		
Central Prison	981	866 (88.28)	115 (11.72)		
Local Prison	337	292 (86.65)	45 (13.35)		
Maximum security	49	39 (79.59)	10 (20.41)		
Medium security	751	654 (87.08)	97 (12.92)		
**Injecting Drug Use**				2.44	0.118*
No	743	660 (88.83)	83 (11.17)		
Yes	1693	1465 (86.53)	228 (13.47)		
**Time in prison for current sentence/remand**				1.55	0.461*
<1 year	782	682 (87.21)	100 (12.79)		
1–5 years	1310	1135 (86.64)	175 (13.36)		
>5 years	350	312 (89.14)	38 (10.86)		
**Region**				9.17	0.329*
Ashanti	567	509 (89.77)	58 (10.23)		
B. Ahafo	215	190 (88.37)	25 (11.63)		
Central	177	147 (83.05)	30 (16.95)		
Eastern	879	766 (87.14)	113 (12.86)		
Gt Accra	140	119 (85.00)	21 (15.00)		
Northern	74	65 (87.84)	9 (12.16)		
Upper west	65	53 (81.54)	12 (18.46)		
Volta	225	194 (86.22)	31 (13.78)		
Western	100	86 (86.00)	14 (14.00)		

*p-values obtained with Pearson’s Chi-squared test.

+p-values obtained with Fisher’s exact test.

### Binary logistic regression models of factors associated with HBV status among inmates

Compared to inmates with no formal education, the adjusted odds of HBV positivity was 35% less (AOR: 0.65, 95% CI: 0.45–0.95, p = 0.024) among inmates with primary level of education. Also, compared to inmates who were underweight, the adjusted odds of HBV positivity was 49% less (AOR: 0.51, 95% CI: 0.27–0.99, p = 0.046) among inmates who were obese.

### HBV status and sexual behavioural characteristics

This study examined the relationship between selected sexual risk behaviours of inmates before and during incarceration and being tested positive for hepatitis B infection. Table 6 shows that none of the sexual behavioural characteristics were significantly associated with hepatitis B infection, though varied proportions were reported. For instance, almost equal proportions of inmates had or had not engaged in casual sex partners (12.6% vs. 13.1% respectively). However, in terms of risk, those who reported they never used condoms in their casual sex engagements were least infected with hepatitis B (12%) as opposed to those who used condoms consistently (15.3%). A similar result was found for sex with CSWs and condom use, where inmates who reported consistent condoms ware most infected with hepatitis B (Table 6).

**Table 6:** Association between HBV status and sexual behaviour variables.

Characteristic	Total	% HBV Positive	p-Value
**Casual sex partners before prison**			*p* = 0.719
Yes	1352	12.6	
No	1087	13.1	
**Number of casual sex partners in the past 12 months**			*p* = 0.383
Single partner	676	13.5	
2 or more partners	657	11.9	
**Condom use during casual sex**			*p* = 0.550
Never use condom	791	12.0	
Inconsistence use	372	13.2	
Consistence use	131	15.3	
**Sex with commercial sex worker (CSW) before prison**			*p* = 0.623
Yes	515	13.4	
No	1923	12.6	
**Frequency of sex with CSW**			*p* = 0.092
At least once a week	121	10.7	
Few times a month	200	17.5	
Few times a year	178	10.7	
**Frequency of condom use with CSW**			*P = 0*.*568*
Never used condom	128	11.7	
Inconsistence use	97	11.3	
Consistence use	263	14.8	
**Heard of inmates being forced to have penetrative sex**			*p* = 0.318
Yes	1285	13.4	
No	1130	11.9	
Don’t know	19	21.1	
**Frequency of forced penetrative sex**			*p* = 0.071
Every few days	329	16.7	
Every few weeks	344	13.4	
Every few months	566	11.8	

### HIV and HBV coinfection

Only 5 of the 2,435 that had both HIV and HBV conclusive test results were both HIV and HBV positive representing 0.21%. Hence a 95% confidence interval of both HIV and HBV positivity in prisons in Ghana is estimated from 0.07% to 0.48%.

## Discussion

Generally, the results of this study suggest significant HIV and HBV infections in prisons in Ghana. To our knowledge, this is the first nationally representative bio-behavioural survey of HIV and HBV infections and other key health problems of prison inmates in all categories of prisons across Ghana. According to this study, the overall HIV and HBV prevalence among prison inmates in Ghana in the year 2013 was 2.38% and 12.82%, respectively, with vast variation based on region and sex. There generally seems to be a decreasing trend with increasing age with regards to HBV prevalence. However, with HIV infections, no such observation was made albeit with much lower prevalence rates. Similar numerical HIV prevalence rates were seen in the general population (1.3%) as compared to the male prison inmates (1.5%). However, a much higher prevalence than in the general population was observed in the female prisoners (11.8%) as compared to the general population [25]. The latter may be accounted for by the higher national prevalence in women than men, and an also smaller population size than the male inmates.

The study provided detailed HIV prevalence within prisons in the various geographical locations in Ghana. Brong Ahafo region (4.67%) and Central region (3.39%) recorded the highest HIV prevalence which was numerically higher than what pertains in the general population of these two regions [26,27]. The Northern region on the other hand recorded the lowest (0%) which correlates with the <1% prevalence in its general population [26]. The HBV prevalence as represented by the presence of HBsAg was numerically similar to the estimated national prevalence of 12.3% [28]. Systematic reviews and meta-analysis done covering the same time period estimated the national prevalence to be 10.2% [28,29]. The current study also provides data to fill national gaps on HBV infection in the Volta (now spilt into Oti and Volta), Western, Upper East and Upper West regions [28,29]. Trends for HBsAg infections are generally expected to reduce with infant vaccination which began in 2002 [30]. This does not seem to reflect in the data obtained for this study. Those within the 18–19 year group had relatively higher HBsAg prevalence than the than the other age groups. Since few HBsAg positive children were seen in a study in children <5 years done in 2013 [31], it is possible that these male inmates may have had low vaccination coverage. Alternately, anti-HBs may have waned for HBV infection to occur.

In contrast to HIV, those with HBV infections may agree with data obtained from different population in Ghana [28]. These may suggest that there may have been similar rates of HIV and HBV infections within the prisons as compared to the general population. This is at variance with a previous study done about a decade ago, which suggested that HIV seropositivity was significantly associated with HBsAg detection in Ghanaian prisoners [32]. The high prevalence seen in that study seems not to have been replicated in this study. It is important to note that the assays used were not the same as what was used this study [28]. On the whole, HIV and HBV infections in prisons may have reduced between 2005 to 2013 based on the data acquired from this study and another conducted by Adjei et. al. [3]. The reduced HIV prevalence determined by this study may also have been affected by sample size as seen in a fairly recent study in Zambia [33].

Generally, there seems to be comparatively limited data on HBV infections in correctional institutions in Africa. On the other hand, HIV prevalence in African prisons has been generally high with up to a prevalence of 17.7% in South African prisons [20,34]. However, a much more recent study which systematically assessed publications on HIV prevalence among inmates from four different African countries reported an average prevalence of 6% [35]. This may suggest that HIV prevalence in correctional facilities on the continent and in Ghana, may be on the decline [20,23,33,35,36].

A randomised sample of 600 prisoners conducted in Senegal reported an HIV prevalence which was four times higher than the national rate [23]. Prisoners enrolled in this study have had sexual encounters while in incarceration (97.8%) with 17.8% being in multiparter relationships. Only 4.6% of the prisoners who were drug users before incarceration admittedly were still on drugs after imprisonment [23]. There is no doubt that high-risk behaviours are a key contributing factor in the spread of blood-bourne viruses such as HIV and HBV in correctional facilities as suggested by several studies around the world [17,18,22,23,37]. However, the absence or low incidence of such high-risk behaviours have also been evidently associated with a reduced prevalence of HIV in prisons based on our study. Even though HIV and HBV have similar routes of transmission [38–40]. For both HIV and HBV infections, exposure to forced penetrative sex approached significance (p = 0.071). In addition, individual inmates hearing about other inmates being forced for penetrative sex was significant for HIV but not HBV infections. It seems that the issue of forced sex is a problem in the prisons but the inmates may not have truthfully responded to the questionnaire for due to the cultural disdain for such practices. This may explain why being female and staying in the prison for more than 5 years increases the risk of having HIV infections. These observations have been made in other studies and may not be new to the prison environment [41]. Feeding in prisons in low to middle income countries is a chronic problem [42]. With increasing length of stay therefore, inmates are likely to become emaciated. This may be associated with low immunity and less ability to fight disease. It is therefore not surprising that those who were without formal education and had stayed longer in prison were those who were likely to have HBV infection. The outcomes of the effects of the sexual behavioral variables on HIV and HBV infections and the prevalence rates therefore suggests that these two viruses may still be transmitted within the prison but not to the extent resulting in very high prevalence as previously seen [3]. Perhaps, the movement of remand prisoners may partly account for the observation. There is evidence to supporting the existence of high-risk activities that fuel the transmission of HIV in prisons in other jurisdictions. For example IDU is a key route of HIV transmission in prisons [38], but there was no significant evidence of IDU use in Ghana’s prison. The most common drugs used in the prisons were marijuana and valium [43]. HIV was higher in the maximum-security prison where most freedoms and contacts between and among inmates are restricted, than in the local and central prisons. However, there was no evidence to conclude that these inmates were infected within incarceration. Further tests are needed to confirm how long HIV positive inmates have been living with the virus, to be able to establish whether inmates were infected prior to imprisonment or not.

Voluntary HIV and HBV testing could be offered new inmates to identify those infected and to offer them the help and support needed. It may be necessary to institute and intensify care and support programs for HIV and HBV in the prisons to reduce spread within the prisons and less likely transmission when they are discharged. As part of the 95-95-95 plan to eradicate HIV [44], self-testing may have to be introduced in the prisons for HIV, and mandatory HBV vaccination for those who are entering. With a median age of 31 years in means that incarceration may be more associated with the young. Those who were vaccinated at birth as part of Ghana EPI programme will therefore be edging towards the median age which may hopefully reduce those susceptible for HIV infections. Health education in prisons should address the modes of transmission of HIV and HBV.

## Conclusion

At the time of the study, the HBV prevalence in the prisons was almost similar to that of the general population. This suggests that the level of transmission within the prisons then may not have resulted in high transmission patterns different from the general population. Nevertheless, the issue of forced penetrative sex must be addressed and appropriate care programmes instituted to protect and treat inmates with HIV and HBV infections. Instituting screening at entry to create a database may be of help to the country.
